# Restoration of antibiotic associated diarrhea induced gut microbiota disorder by using *Dictyophora indusiata* water-insoluble polysaccharides in C57BL/6J mice

**DOI:** 10.3389/fnut.2025.1607365

**Published:** 2025-07-10

**Authors:** Yong Lai, Qifan Zhang, Maohan Xu, Xiurong Guo, Quan Zhou, Qiuyu Liu, Huiling Deng, Can Song

**Affiliations:** ^1^School of Pharmacy, Southwest Medical University, Luzhou, Sichuan, China; ^2^Institute of Traditional Chinese Medicine, Sichuan Academy of Chinese Medicine Sciences, Chengdu, Sichuan, China; ^3^Chongqing Key Laboratory of Prevention and Treatment for Occupational Diseases and Poisoning, The First Affiliated Hospital of Chongqing Medical and Pharmaceutical College, Chongqing, China

**Keywords:** polysaccharides, antibiotic-associated diarrhea, gut microbiota, *Dictyophora indusiata*, prebiotic

## Abstract

**Background:**

A healthy gut depends on a balance of commensal and probiotic bacteria, and prolonged and inappropriate clinical use of antibiotics can cause an imbalance in the gut flora, resulting in antibiotic-associated diarrhea (AAD). Macrofungal polysaccharides are rich in bioactivities and have attracted much attention for their good performance in anti-inflammatory, antioxidant, anti-obesity and anti-tumor properties. Therefore, we explored the role of *Dictyophora indusiata* water-insoluble polysaccharides (DIPY) in modulating the gut flora to improve AAD.

**Methods:**

We initially prepared the water-insoluble polysaccharides derived from *Dictyophora indusiata*. Subsequently, by comprehensively evaluating multiple parameters including the body weight, dietary patterns, cecal histomorphological characteristics, intestinal microbiota composition, concentrations of short-chain fatty acids (SCFAs), and the levels of inflammatory factors in the antibiotic-associated diarrhea (AAD) model animals, we delved into both the action mechanisms of these polysaccharides and their impacts on the intestinal flora and metabolites within C57BL/6J mice.

**Results:**

Our results showed that DIPY effectively ameliorated AAD in mice by modulating the intestinal flora, increasing microbial diversity, the Shannon and Ace index was significantly higher in the DIPY group than in the NR group after the DIPY intervention (*p* < 0.001). Also, compared with the NR group, DIPY increasing the relative abundance of *Parasutterella* and *Blautia*, increasing the production of acetic acid (*p* < 0.001), and decreasing the levels of LPS, MCP-1, TNF-*α*, and IL-6 (*p* < 0.05) which attenuating inflammatory responses.

**Conclusion:**

This study demonstrated that DIPY has intestinal prebiotic function, which provides a basis for further development of functional products for the treatment of AAD.

## 1 Introduction

Human health maintains an intricate relationship with the enteric microbial ecosystem, where symbiotic interactions between gut microbiota and their mammalian hosts constitute a fundamental determinant of energy metabolism and physiological homeostasis (1). The gut microbiota consists of trillions of diverse microbes that perform essential functions like barrier protection, nutrient synthesis, metabolism regulation, and immune modulation. This complex network is termed a “metabolic powerhouse” and “second genome” in biomedical research (2). Physiological equilibrium in the host organism fundamentally depends on maintaining microbial homeostasis, with intestinal dysbiosis frequently serving as a precursor to pathological manifestations (3). Antibiotic-associated diarrhea (AAD) represents a gastrointestinal complication characterized by diarrheal symptoms and gut microbial dysregulation triggered by therapeutic antibiotic administration (4). This condition arises from the dual pharmacological action of antibiotics: while effectively eliminating pathogenic bacteria, these agents may concurrently destabilize the intestinal microbiome’s ecological equilibrium. Notably, Broad-spectrum antibiotics are more likely to cause AAD because they indiscriminately target microbes. The severity of AAD depends on the antibiotic’s activity range, treatment duration, and disruption of gut microbiota-host interactions (5). The incidence of AAD exhibits significant variability contingent upon the specific population under consideration and the class of antibiotics administered. For instance, in high-risk populations, which may include the elderly, immunocompromised individuals, and those with pre-existing gastrointestinal disorders, the incidence of AAD can soar to a staggering 36.79% (6). With the continuous enhancement of people’s living standards and the concomitant rise in healthcare utilization, the frequency of antibiotic prescriptions has also increased. This, in turn, has led to a progressive exacerbation of the AAD problem. The over-use and misuse of antibiotics not only disrupt the delicate balance of the gut microbiota but also heighten the risk of AAD occurrence. As such, there is an immediate and pressing need to explore novel and effective strategies.

In recent years, with the deepening of research on gut microecology, people have had a more profound understanding of its crucial role in human health. Against this backdrop, the relationship between antibiotic use and gut health has gained increasing attention (7). Meanwhile, emerging therapies like probiotics and prebiotics have emerged, opening up new avenues for the treatment and prevention of intestinal diseases, such AAD (8). Some studies have demonstrated that consuming polysaccharide prebiotics can significantly modulate the composition and diversity of gut microbiota, helping maintain intestinal microecological balance, and thus promoting gut health (9). For example, purple sweet potato polysaccharides can increase the diversity of gut microbiota in AAD mouse models, reduce inflammation levels and cecum index, improve ileal tissue morphology, and increase the content of short-chain fatty acids (SCFAs), indicating their prebiotic potential (10). Similarly, bamboo shoot polysaccharides have been proven to promote the growth of beneficial bacteria such as Firmicutes, Lactobacillus, and Lachnospiraceae_NK4A136_group, and enhance their metabolic synthesis of SCFAs (11). Polysaccharides are macromolecular polymers composed of more than ten monosaccharide units linked by glycosidic bonds, widely present in nature and serving as integral components of the structure and function of many organisms (12). As natural functional prebiotics, polysaccharides generally exhibit the characteristic of non-uniform degree of polymerization. Meanwhile, at the molecular level, they demonstrate stereochemical diversity formed by different sugar units, various linkage modes, and complex branching structures. Therefore, they possess multiple biological activities such as antioxidant, anticancer, anti-inflammatory, lipid-lowering, and hypoglycemic effects, and have shown remarkable efficacy in regulating the intestinal microecology (13).

As a prominent edible and medicinal macrofungi, *Dictyophora indusiata* boasts remarkable nutritional value. Its rich nutrients and diverse physiological activities have drawn extensive attention (14). Modern scientific analysis reveals that *Dictyophora indusiata* contains numerous bioactive components beneficial to humans. Among them, proteins, amino acids, polysaccharides, vitamins, and trace elements are the major active substances, holding great research and application potential (15). Among the many active components of *Dictyophora indusiata*, its polysaccharides have gained prominence due to their significant bioactivity. They offer various health-beneficial functions, such as antioxidant, anticancer, anticoagulant, anti-inflammatory, immune-modulating, and blood-glucose-lowering effects. These functions endow *Dictyophora indusiata* polysaccharides with potential for preventing and adjunctively treating multiple chronic diseases. Common preparation methods include hot-water extraction, alkali extraction, ultrasound, and enzymatic hydrolysis (16). Water-soluble polysaccharides from *Dictyophora indusiata* restore antibiotic-induced gut dysbiosis by promoting beneficial bacteria growth while reducing endotoxins and inflammation (17). Another study focused on water-insoluble polysaccharides from *Poria cocos*. Results showed that this water-insoluble polysaccharide could effectively alleviate lincomycin hydrochloride-induced AAD. It also mitigates inflammatory responses by promoting SCFAs production (18). Therefore, *Dictyophora indusiata* water-insoluble polysaccharides are expected to become a novel, efficient prebiotic product for AAD relief. Still, there are many unknown details and mechanisms in this field worthy of further exploration.

In this experiment, we first prepared *Dictyophora indusiata* water-insoluble polysaccharides and then explored their effects on the intestinal flora and metabolites in C57BL/6J mice by assessing the body weight diet, cecum histomorphology, intestinal flora, SCFAs, and levels of inflammatory factors in AAD model animals.

## 2 Materials and methods

### 2.1 DIPY preparation and animal experiments

The preparation and extraction of *Dictyophora indusiata* water-insoluble polysaccharides was carried out referenced to our team’s previous study (19), the concise description is as follows: alkali extraction, acid precipitation and purification, that is, soaking the water extraction residue of *Dictyophora indusiata* in 0.5 mol/L NaOH solution for 4 h, repeating three times to obtain a crude polysaccharide alkali solution, adding 0.5 mol/L hydrochloric acid to neutralize and precipitate the polysaccharide, centrifuging and washing, and then removing impurities through a cellulose dialysis bag to finally obtain DIPY.

The Ethics Committee of the Animal Experimentation Center of Southwest Medical University approved this animal experiment procedure. After 1 week of acclimatization in an environment with a temperature of 22–25°C and a relative humidity of 50–55%, 21 male mice (6 weeks old, the average body weight is 20–22 g, license: SCXK [Jing] 2024–0001) were randomly and equally divided into 3 groups of 7 mice each, namely, a blank control group (CN), a natural recovery group (NR) and a polysaccharide gavage group (DIPY) for antibiotic-associated diarrhea. The whole animal experiment was divided into two phases: pathological model phase and recovery phase. The CN group was continuously gavaged with Saline, but NR and DIPY groups were first gavaged with 3 g/kg lincomycin hydrochloride twice a day for 3 days, which is pathological model phase (20). Successful AAD modeling was indicated by the presence of soft, watery, and mucous feces in the mice (21). Subsequently, mice in the DIPY group were gavaged with 300 mg/kg/day polysaccharide for 1 week, and the NR was left untreated, which is recovery phase. Mouse body weight, dietary water intake, and diarrhea status scores were recorded continuously, and mouse feces, blood, and cecum tissues were collected, rapidly frozen in liquid nitrogen, and then transferred to a − 80°C refrigerator for subsequent experimental measurements.

### 2.2 HE and AB/PAS staining

HE staining (Hematoxylin–eosin staining) can be used to observe the morphology and structure of the cell interior, hematoxylin can bind to the chromatin and nucleic acids inside the cell with a purple-blue color; eosin can bind to the cell matrix with a red color. AB/PAS stain (Alcian blue/periodic acid-Schiff stain) is used in histology to detect sugars in tissues, also known as glycogen staining, and can be used to show glycogen, neutral mucus substances and certain acids. Briefly, blind tissues were fixed in formalin and paraffin-embedded. Paraffin-embedded sections were deparaffinized with xylene, then rehydrated with ethanol, stained with hematoxylin and AB/PAS to observe histologic changes in the cecum.

### 2.3 16Sr RNA microbiota analysis

Total DNA was extracted from the samples using the DNA Fecal Kit. The variable V3–V4 regions of genes were amplifed using primers, 16 s-F (5′-AGAGTTTGATYMTGGCTCAG-3) and 16 s-R (5-TGCTGCCTCCCG PCZGGAGT-3′). The products were purified to remove unbound primers, enzymes, and other impurities and then sequenced on the Illumina MiSeq platform. Sequencing data were processed and analyzed using QIIME2, and sequences with 100% similarity were classified as amplicon sequence variants (ASVs). Samples sequencing was supported by Majorbio Bio-Pharm Technology Co., Ltd. (Shanghai, China). All gene sequencing raw sequence data are stored in the NCBI Sequence Read Archive (SRA) under the accession number PRJNA1007402.

### 2.4 Targeted SCFAs microbiota analysis

Gas chromatography–mass spectrometry (GC–MS) was used to determine the concentration of SCFAs in mouse fecal samples. A gas chromatography-mass spectrometer is an analytical instrument that combines gas chromatography (8) and mass spectrometry (22). Its principle is as follows: after the sample is separated into individual components by GC, it enters the MS for ionization and mass analysis. Through the matching of mass-to-charge ratio (m/z) and spectral libraries, the components of the substance can be quickly identified. Fresh mouse feces were first collected in animal experiments and quickly frozen and stored. A series of short-chain fatty acid standards with known concentrations were prepared for the construction of calibration curves and method validation. 20 mg of fecal sample was weighed and added to 800 μL of 0.5% phosphoric acid in water. The sample was frozen and ground for 3 min, sonicated for 10 min, centrifuged for 15 min, then extracted with 200 μL of n-butanol, sonicated and centrifuged again, and then prepared for analysis on the machine. The SCFAs in the samples were separated using a gas chromatography system, and the separated SCFAs were detected by mass spectrometry (22) with scanning in selected ion scan mode. The analytical instrument for this experiment was a gas-mass spectrometer. Finally, the quantitative results were statistically analyzed, and the default parameters of MassHunter quantitative software were used for automatic identification and integration of each ionic fragment of the target SCFAs. The detected concentration of each sample was calculated through the standard curve, which was converted to the actual content of SCFAs in the samples.

### 2.5 Elisa assay analysis

Mouse LPS, MCP-1, TNF-*α* and IL-6 ELISA kits were sourced from ZCIBIO Technology Co., Ltd. (Shanghai, China). The commercial kits use sandwich method enzyme-linked immunosorbent assay (ELISA), and the experimental operation was carried out according to the instructions of the kits, and the absorbance (OD value) was measured at 450 nm with an enzyme meter to calculate the concentration of the samples.

### 2.6 Statistical analysis

Data from all analyses are expressed as mean ± standard error (8). Statistical methods (e.g., *t*-test, ANOVA, Wilcoxon test, etc.) were used to detect significant differences in microbial composition between groups, and to predict the functional properties of the microbial community by PICRUSt2. Data from the Elisa experiment were statistically analyzed using Prism 10.1.2 software, and the significant differences in SCFAs data were tested using the independent *t*-test. Values of * *p* < 0.05 were considered statistically significant.

## 3 Results

### 3.1 DIPY improves AAD symptoms in C57BL/6J mice

**Figure 1:**
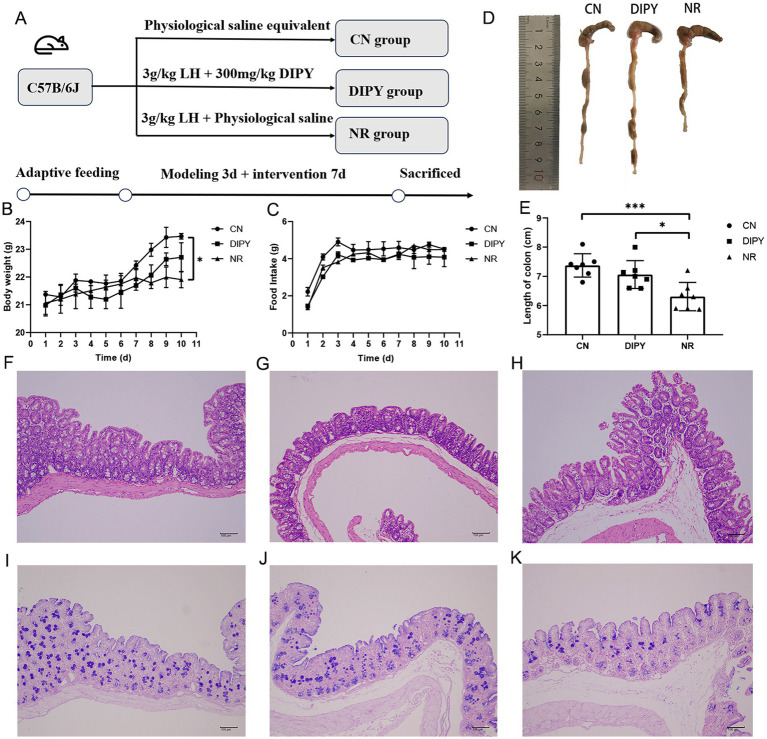
Animal experimental design and animal experimental results. **(A)** The process of designing animal experiments. **(B)** The weight changes of mice. **(C)** The changes in food consumption of mice. **(D)** The comparison of the cecum in mice. **(E)** The average length of the cecum in mice. **(F)** HE staining of the mice cecum in CN. **(G)** HE staining of the mice cecum in NR. **(H)** HE staining of the mice cecum in DIPY. **(I)** AB/PAS staining of the mice cecum in CN. **(J)** AB/PAS staining of the mice cecum in NR. **(K)** AB/PAS staining of the mice cecum in DIPY.

The procedure of the AAD animal experiment is shown in Figure 1A. After 3 days of continuous gavage of 3 g/kg lincomycin hydrochloride, the feces of the mice became wetter and softer, indicating the success of the creation of the antibiotic-associated diarrhea model. After continuous gavage of DIPY for the following week, mice in the DIPY group gained weight and mice in the NR group lost weight compared with the CN group (Figure 1B). Meanwhile, the mice in the NR group had a significant decrease in diet compared with the remaining two groups (Figure 1C). In the comparison of the mean length of mouse cecum (Figures 1D,E), which was reduced in the NR group compared to the DIPY and CN groups, the treatment of polysaccharides significantly increased the length of mouse cecum.

Observing the histological changes of HE staining in the three groups, the cecum tissue of the NR group showed inflammatory cell infiltration, mucosal defects and rupture of epithelial cells (Figure 1G). The histological characteristics of the CN group and the DIPY group were as shown in Figures 1F,H, which showed that their intestinal mucosa was more intact, and the epithelial cells were arranged in a regular pattern. AB/PAS staining (Figures 1I–K) showed a reduced number of cup cells, reduced crypt structures and mucus in the NR group compared to the DIPY and CN groups.

### 3.2 DIPY regulates the structure and composition of the gut microbiota

**Figure 2:**
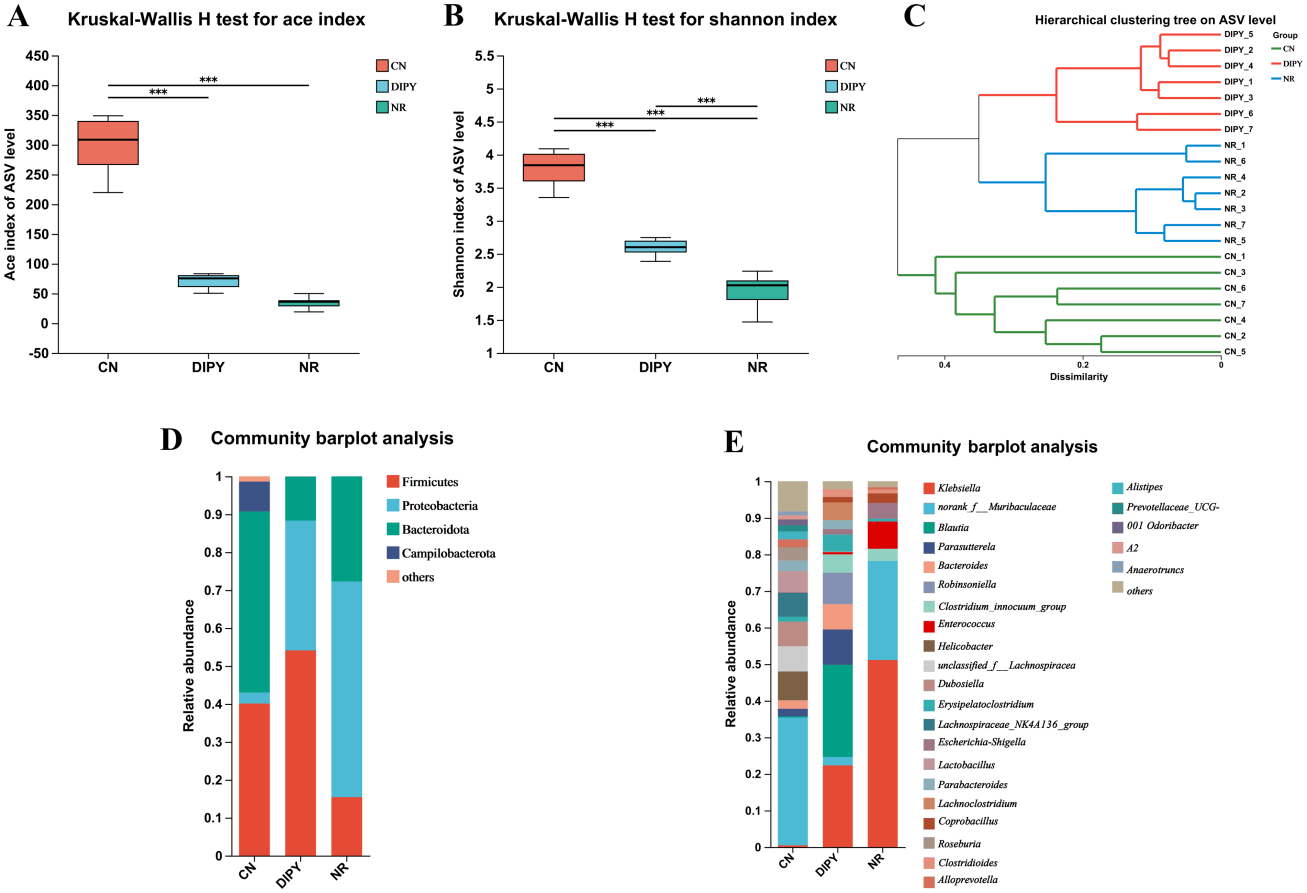
Results of diversity and composition of intestinal flora in mice. **(A)** Kruskal-Wallis H test for ace index. **(B)** Kruskal-Wallis H test for Shannon index. **(C)** Hierarchical clustering tree on ASV level. **(D)** Community barplot analysis of three groups at the phylum level. **(E)** Community barplot analysis of three groups at the genus level.

In this experiment, we examined the diversity of the intestinal flora of mice, which is an important indicator of the complexity of microbial communities, including *α*-diversity and *β*-diversity. The α-diversity of the intestinal microbial community refers to the abundance and homogeneity of microbial species in the intestine, the α-diversity statistics are summarized in Supplementary Table S1. As shown in Figures 2A,B, the direct use of antibiotics killed some of the gut bacteria, causing a decrease in diversity, and the Ace and Shannon indices were significantly higher in the DIPY group than in the NR group after the DIPY intervention (*p* < 0.001).

Hierarchical Clustering Tree (HCT) of gut flora can be used to describe and analyze microbial community structure by grouping microbial samples based on their similarities. As shown in Figure 2C, each node represents a cluster and the branches of the tree indicate the similarity between the samples in the tree, The results showed different distributions in the 3 groups and the clustering of the DIPY and NR groups indicated that antibiotic use caused the most significant differences. As shown in the community barplot analysis in Figures 2D,E, the main intestinal flora at the phylum level includes Firmicutes, Proteobacteria, Bacteroidota, Campilobacterota, and others; and the main intestinal flora at the genus level include *Klebsiella*, norank_f__*Muribaculaceae*, *Blautia*, *Parasutterella, Robinsoniella,* and others.

**Figure 3:**
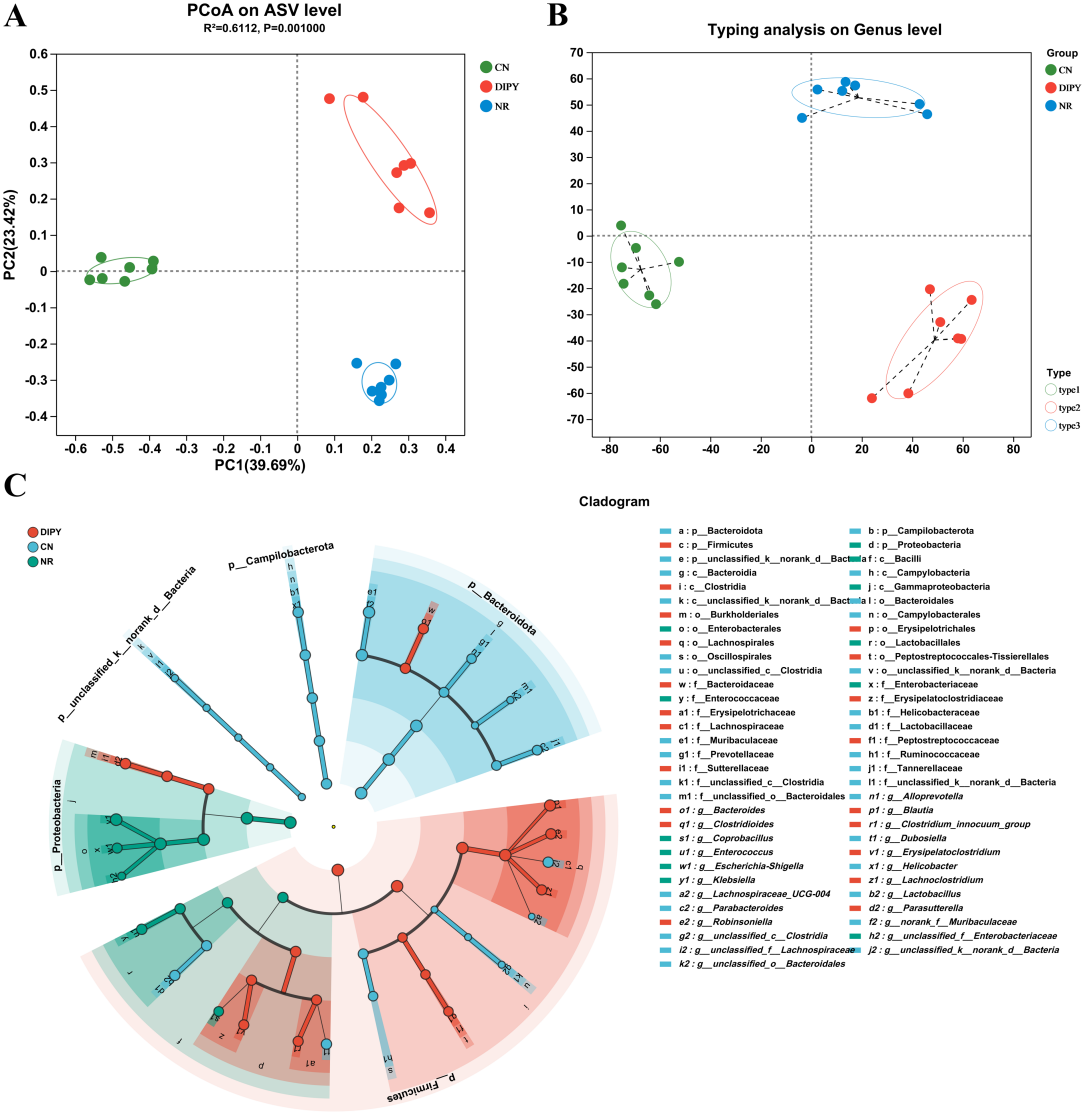
Microbial community structure and composition. **(A)** PCoA on ASV level. **(B)** Typing analysis on genus level. **(C)** The cladogram of Lefse analysis.

*β*-diversity refers to differences in microbial community composition between individuals or samples. We performed principal co-ordinates analysis (PCoA) on ASV level and typing analysis on genus level, to visualize the β-diversity of microbial communities in different groups (Figures 3A,B). As can be seen from the matrix, the CN group is clustered on the left side, while the samples in the DIPY and NR groups are distributed in the upper and lower right regions, respectively, and there is a significant difference between the mice in the experimental and control groups. In addition, LefSe (Linear Discriminant Analysis Effect Size) is a statistical method for microbiome data analysis that centers on the calculation of Linear Discriminant Analysis scores for assessing the variability of microbial taxa across groups (Figure 3C). In the LEfSe bar chart, there are 31 key differential species in the CN group, among which *Bacteroidota* and *Muribaculaceae* are the most significant ones. The DIPY group has 19 key differential bacterial species, with *Clostridia* and *Lachnospirates* being most relatively prominent. In contrast, the NR group only has 11 key differential species, and the most significant bacterium is *Enterobacterales* (Supplementary Figure S1).

### 3.3 DIPY promotes the abundance of beneficial intestinal bacteria

**Figure 4:**
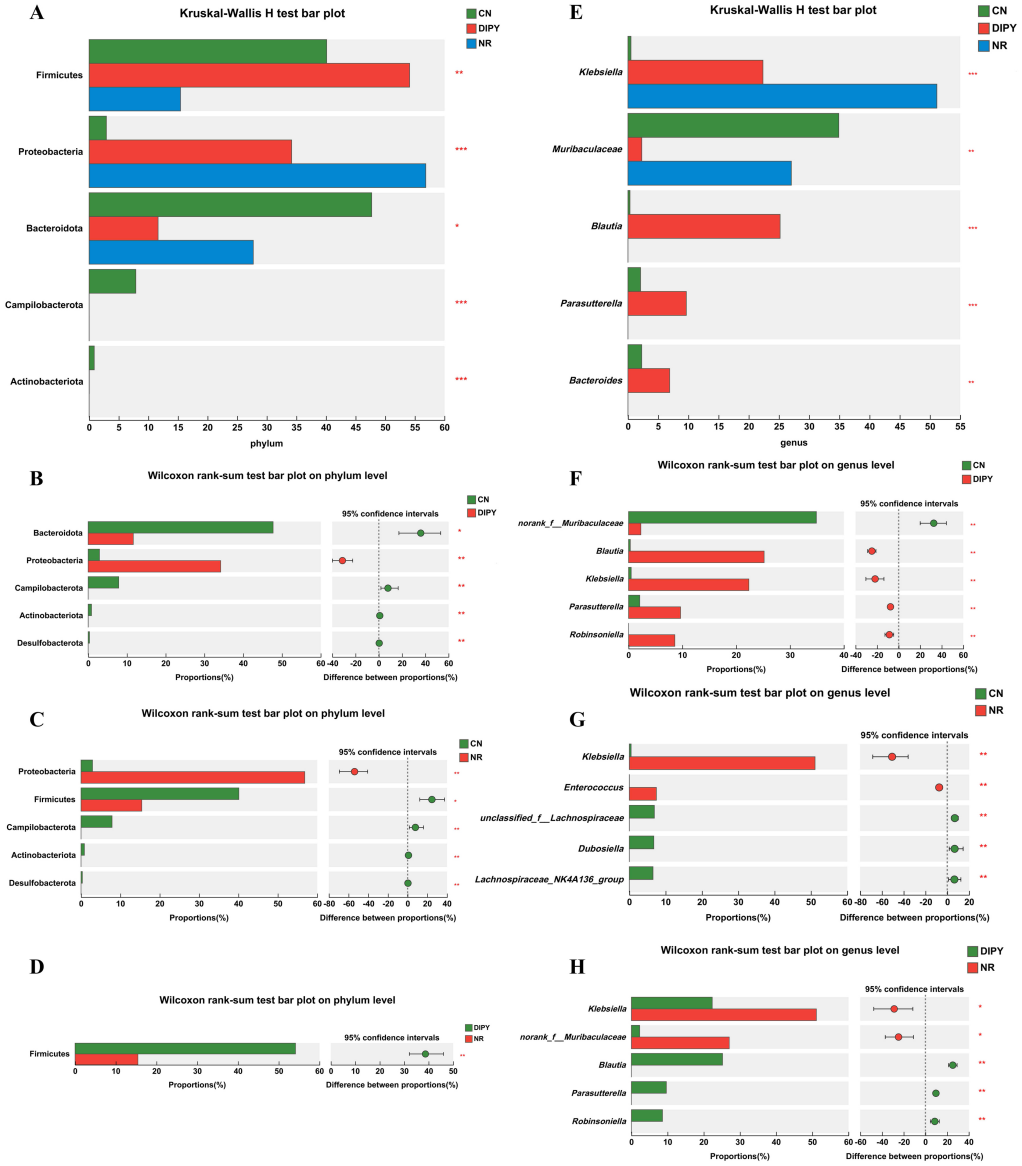
Results of differences in microbial taxa among multiple groups. **(A)** The Kruskal-Wallis H test bar plot of three groups on the phylum level. **(B)** The Kruskal-Wallis H test bar plot of CN and DIPY. **(C)** The Kruskal-Wallis H test bar plot of CN and NR. **(D)** The Kruskal-Wallis H test bar plot of NR and DIPY. **(E)** The Kruskal-Wallis H test bar plot of three groups on the genus level. **(F)** The Kruskal-Wallis H test bar plot of CN and DIPY. **(G)** The Kruskal-Wallis H test bar plot of CN and NR. **(H)** The Kruskal-Wallis H test bar plot of NR and DIPY.

In 16Sr RNA high-throughput sequencing, we used the Kruskal-Wallis test for significant microbial taxa in multiple-group comparison and used the Wilcoxon rank-sum test to determine if these microbial taxa differed in group comparisons. As seen in Figure 4A, the Kruskal-Wallis H test bar plot at the phylum level showed that Firmicutes, Proteobacteria, Bacteroidota, Campilobacterota and Actinobacteriota had significant differences in the 3-group comparison. In Wilcoxon rank-sum test bar plot on phylum level between groups (Figures 4B–D), mice in the NR group that used lincomycin hydrochloride significantly decreased the relative abundance of Firmicutes and increased the relative abundance of Proteobacteria. In contrast, mice in the DIPY group using polysaccharides significantly increased the relative abundance of Firmicutes and decreased the relative abundance of Bacteroidota. As seen in Figure 4E, the Kruskal-Wallis H test bar plot at the genus level showed that *Klebsiella, Muribaculaceae, Blautia, Parasutterella*, and *Bacteroides* had significant differences. In Wilcoxon rank-sum test bar plot on phylum level between groups (Figures 4F–H), mice in the NR group using lincomycin hydrochloride significantly increased the numbers of *Klebsiella* and *Enterococcus*, and decreased the numbers of unclassified_f__*Lachnospiraceae, Dubosiella* and *Lachnospiraceae*_NK4A136_group numbers. However, the DIPY group significantly decreased the relative abundance of norank_f__*Muribaculaceae* and increased the relative abundance of *Robinsoniella, Parasutterella*, and *Blautia* compared to the CN and NR groups. The heat map of the functional prediction (Supplementary Figure S2) was obtained based on FAPROTAX. It is clearly evident that DIPY has regulated the composition of microorganisms with different functions in the mouse gut. The phenotypic prediction of the microbiota in the three groups was carried out using the Kruskal-Wallis H test method, as depicted in Supplementary Figures S3A,B, compared with the CN group, the microbiota in the NR group and the DIPY group formed more biofilms and contained more potential pathogenic bacteria. Clusters of Orthologous Groups (COG) serves as a means for differential gene function annotation and plays a significant part in predicting protein functions. The classifications of COG functions are presented in Supplementary Figure S4.

### 3.4 DIPY promotes the concentration of intestinal SCFAs in mice

**Figure 5:**
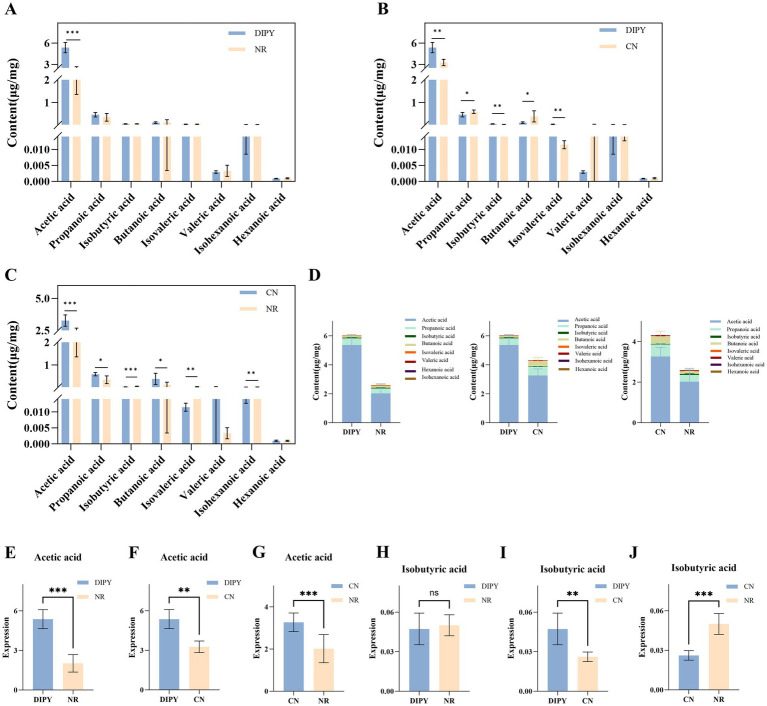
Results of targeted metabolomics detection. **(A)** The bar chart of SCFAs content in NR and DIPY. **(B)** The bar chart of SCFAs content in CN and DIPY. **(C)** The bar chart of SCFAs content in NR and CN. **(D)** The distribution of SCFAs content in three groups. **(E)** The comparation of acetic acid in NR and DIPY. **(F)** The comparation of acetic acid in CN and DIPY. **(G)** The comparation of acetic acid in NR and CN. **(H)** The comparation of isobutyric acid in NR and DIPY. **(I)** The comparation of isobutyric acid in CN and DIPY. **(J)** The comparation of isobutyric acid in NR and CN.

To clarify the potential effects of antibiotics and DIPY on the metabolites of mouse intestinal flora, we employed targeted metabolomics to examine SCFAs in mouse feces and the results are shown in Figure 5. DIPY significantly increased the concentrations of acetic acid compared to the NR group (Figure 5A). The acetic acid levels were higher in the DIPY group than in the NR group (Figure 5B), and in the CN group, the administration of lincomycin hydrochloride reduced the concentration of various SCFAs (Figure 5C). The bar distribution of SCFAs is shown in Figure 5D, which includes acetic acid, propanoic acid, butanoic acid, isobutyric acid, isovaleric acid, isohexanoic acid, valeric acid and hexanoic acid. Acetic acid was the most abundant among all SCFAs and it was significantly higher in the DIPY group than in the NR and CN groups (Figures 5E–G). In addition, in another important and differential butyric acid, isobutyric acid in the CN group was significantly lower in DIPY and NR groups (Figures 5H–J).

### 3.5 DIPY reduces LPS and inflammatory factors in C57BL/6J mice

**Figure 6:**
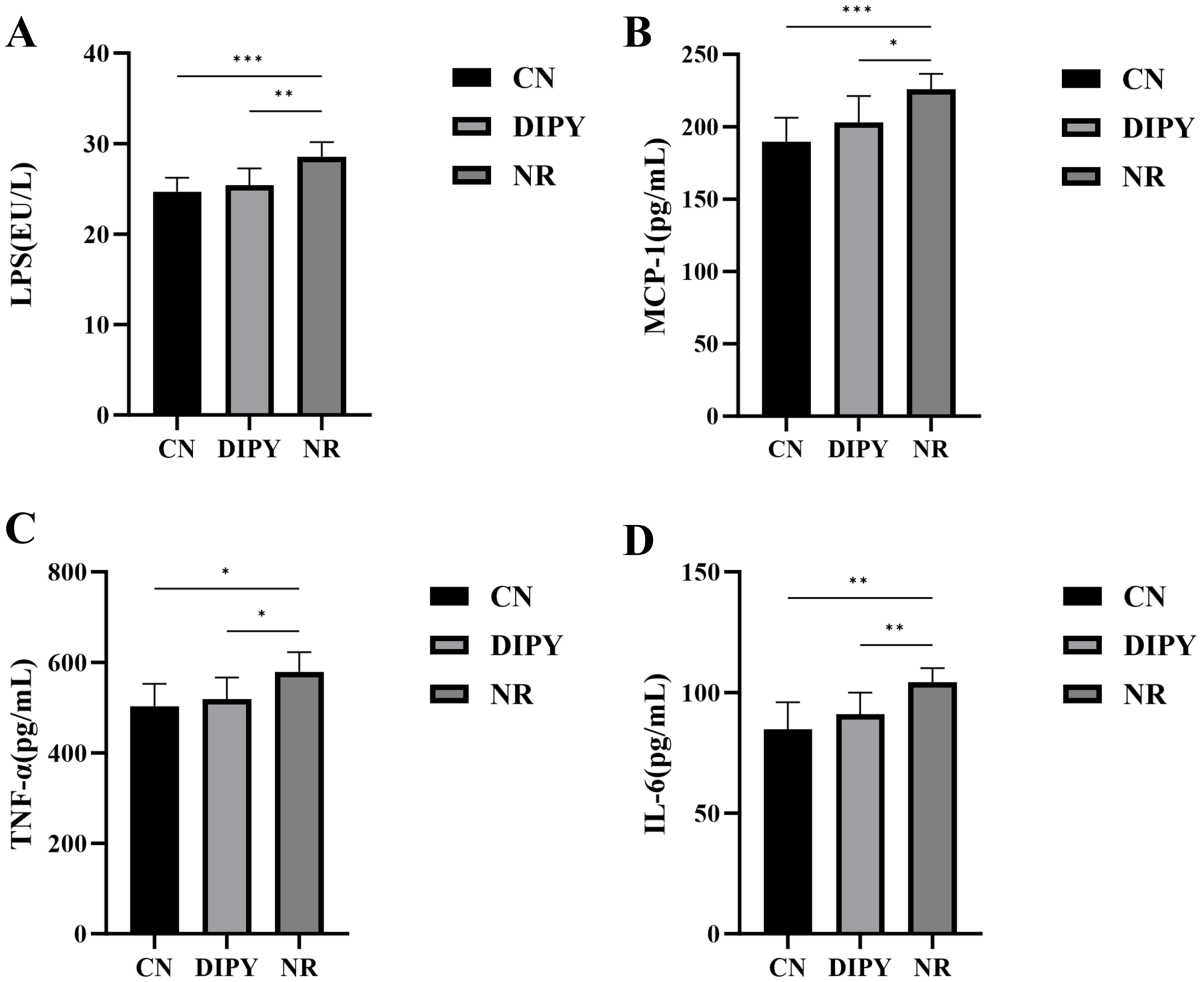
Results of inflammatory factor level changes. **(A)** The comparison of LPS levels in mouse serum. **(B)** The comparison of MCP-1 levels in mouse serum. **(C)** The comparison of TNF-*α* levels in mouse serum. **(D)** The comparison of IL-6 levels in mouse serum.

We determined the concentrations of LPS, MCP-1, TNF-*α* and IL-6 in mouse serum according to the instructions of the kit, and the results are shown in Figure 6. Lipopolysaccharide (LPS) is a major component of the cell wall of Gram-negative bacteria, and the statistical results in Figure 6A showed that the concentration of LPS in mice in the AAD model group was significantly higher compared to the NR group, with a highly significant difference (*p* < 0.05). Compared with the NR group, the concentration of LPS in mice in the DIPY group was reduced, and the difference was significant (*p* < 0.05). The results showed that the serum concentrations of MCP-1, TNF-α and IL-6 were significantly higher in the NR group of mice compared to the CN group, with significant differences (*p* < 0.05). And, after 1 week of treatment, DIPY significantly reduced MCP-1, TNF-α and IL-6 concentrations (*p* < 0.05). These results suggest that administration of DIPY effectively improved the inflammatory response of organismal AAD.

## 4 Discussion

The gut microbiota, as an important area of biomedical research in recent years, have attracted the attention of numerous scientists. These microbial communities not only play a crucial role in maintaining human health but are also closely related to the occurrence and development of many chronic diseases, such as obesity, diabetes, inflammatory bowel disease, and certain types of cancer. Therefore, it is of great significance to maintain the homeostasis and health of the gut microbiota (23). Among the many potential intervention methods, bioactive macromolecules in natural products have shown great application potential, especially in the medical field. Polysaccharides, as a class of long-chain carbohydrates widely present in nature, have shown good application prospects due to their multiple biological activities, including anti-tumor, anti-inflammatory, anti-oxidant, anti-aging and immune regulation and other biological activities (24). Macrofungal polysaccharides have a long history of application in traditional medicine, and recent studies have also confirmed their effectiveness in the prevention and treatment of chronic diseases. These polysaccharides exhibit a great prebiotic potential and deserve further in-depth exploration (25). The polysaccharide of Cereus sinensis exerts its prebiotic effect by increasing the diversity of intestinal microorganisms in mice with AAD and significantly increasing the relative abundance of beneficial bacteria (26). In addition, *Panax ginseng* polysaccharide has been found to successfully counteract the intestinal disturbances caused by lincomycin by increasing the number of probiotic bacteria and regulating energy metabolism (27). *Poria cocos* polysaccharide significantly improves the symptoms of antibiotic-associated diarrhea by increasing the abundance of Lactobacillus and reducing the level of inflammation (28).

Polysaccharides are a type of biological macromolecule. Due to their special chemical structures, they are not easily broken down by digestive enzymes in the host body. This allows polysaccharides to pass smoothly through the stomach and enter downstream parts such as the cecum and colon. In these parts, a rich population of microorganisms starts to play a role, utilizing these undigested polysaccharides for fermentation and metabolism, and thus generating a series of SCFAs, which mainly include acetate, propionate, and butyrate (29). These SCFAs are not only an important energy source for intestinal epithelial cells but also play a crucial role in maintaining the balance of water and electrolytes and promoting intestinal health (30). However, when the body encounters unfavorable factors, such as the use of antibiotics and intestinal inflammation, the balance of the intestinal flora will be disrupted, leading to a decrease in the fermentation activity of the microbiota and a reduction in the concentration of metabolites such as SCFAs (31). This imbalance may trigger a series of health problems, and AAD is a typical example. The occurrence of AAD is closely related to changes in the intestinal flora, and the supplementation of polysaccharide prebiotics can effectively improve this situation. These prebiotics can relieve the symptoms of AAD by increasing the number of probiotics in the intestine, raising the concentration of SCFAs, and reducing the inflammatory response (32). In our experiment, we paid special attention to a kind of water-insoluble polysaccharide derived from *Dictyophora indusiata*. The results showed that it can not only significantly regulate the structure and function of the intestinal flora but also promote the production of metabolites related to the probiotic functions of the intestine, and it can be used to alleviate AAD.

Healthy gut microbiota is in a state of dynamic equilibrium, which is disturbed by the use of lincomycin hydrochloride (33). In animal experiments, antibiotics killed some of the intestinal commensal and probiotic bacteria, such as unclassified_f__*Lachnospiraceae, Dubosiella* and *Lachnospiraceae*_NK4A136_group, and caused a proliferation of harmful and conditionally pathogenic bacteria, such as *Klebsiella* and *Enterococcus*. Previous studies have shown that the status of gut microbiota in AAD is usually characterized by reduced diversity and abundance, a decrease in beneficial flora and an increase in conditionally pathogenic bacteria, which is consistent with our study (22). Interestingly, DIPY ameliorated the symptoms of AAD and significantly increased the relative abundance of *Parasutterella* and *Blautia* at the genus level. Studies have shown that *Blautia* has many potential health benefits, such as *Blautia*’s ability to effectively alleviate obesity and chronic metabolic diseases, and its antimicrobial activity against specific microorganisms (34). In addition, as a major butyrate producer, *Blautia* may also exert anti-inflammatory properties by upregulating the production of regulatory T cells and SCFAs (35). *Parasutterella* is an important member of the gut microbiota and is considered part of the core fecal microbiota of the healthy human gut, playing a role in bile acid maintenance and cholesterol metabolism (36). In our experiments, the water-insoluble polysaccharide of *Dictyostelium indusiata* specifically modulated the number of *Parasutterella* and *Blautia,* suggesting that this is likely to be the target of the polysaccharide’s action in ameliorating AAD.

Different bacterial species in the intestinal flora produce LPS with varying effects on the host, and the use of antibiotics kills a large number of beneficial bacteria and promotes the growth and reproduction of harmful bacteria while producing large amounts of LPS (37). MCP-1 (Monocyte chemoattractant protein-1) is a monomeric polypeptide that is a cytokine that induces leukocyte chemotaxis to sites of inflammation (38). Tumor necrosis factor alpha (TNF-*α*) and interleukin 6 (IL-6) are key cytokines linking inflammation and the immune system, and they participate in the amplification and maintenance of the inflammatory response by promoting the recruitment, activation, and proliferation of inflammatory cells (39). In this experiment, on the one hand, metabolites produced by the intestinal flora, such as SCFAs, can affect the intestinal barrier function and the host’s immune response, which in turn affects the expression of MCP-1, TNF-α and IL-6. On the other hand, AAD-induced dysbiosis of the intestinal flora leads to weakening of the intestinal barrier function, which in turn increases the production of LPS, which can trigger the production of TNF-α and IL-6, further leading to an inflammatory response.

Research on polysaccharides has attracted increasing attention, especially in recent years, studies on the influence of polysaccharide structure on their effects have become a hot topic. On the one hand, some studies have reported that polysaccharides can undergo directional modification of their structures through fermentation to alter structural characteristics, thereby influencing the functional activities of polysaccharides (40, 41), and the type of monosaccharides plays a crucial role in biological activity (42). On the other hand, research has also indicated that molecular weight is a key factor affecting the biological activity of polysaccharides (43). Therefore, establishing a systematic set of characterization methods for water-insoluble polysaccharides in future studies will enable us to gain a deeper understanding of the structure–function relationship of polysaccharides and explore the mechanisms by which polysaccharides regulate the intestinal flora. These aspects warrant further investigation.

**Figure 7:**
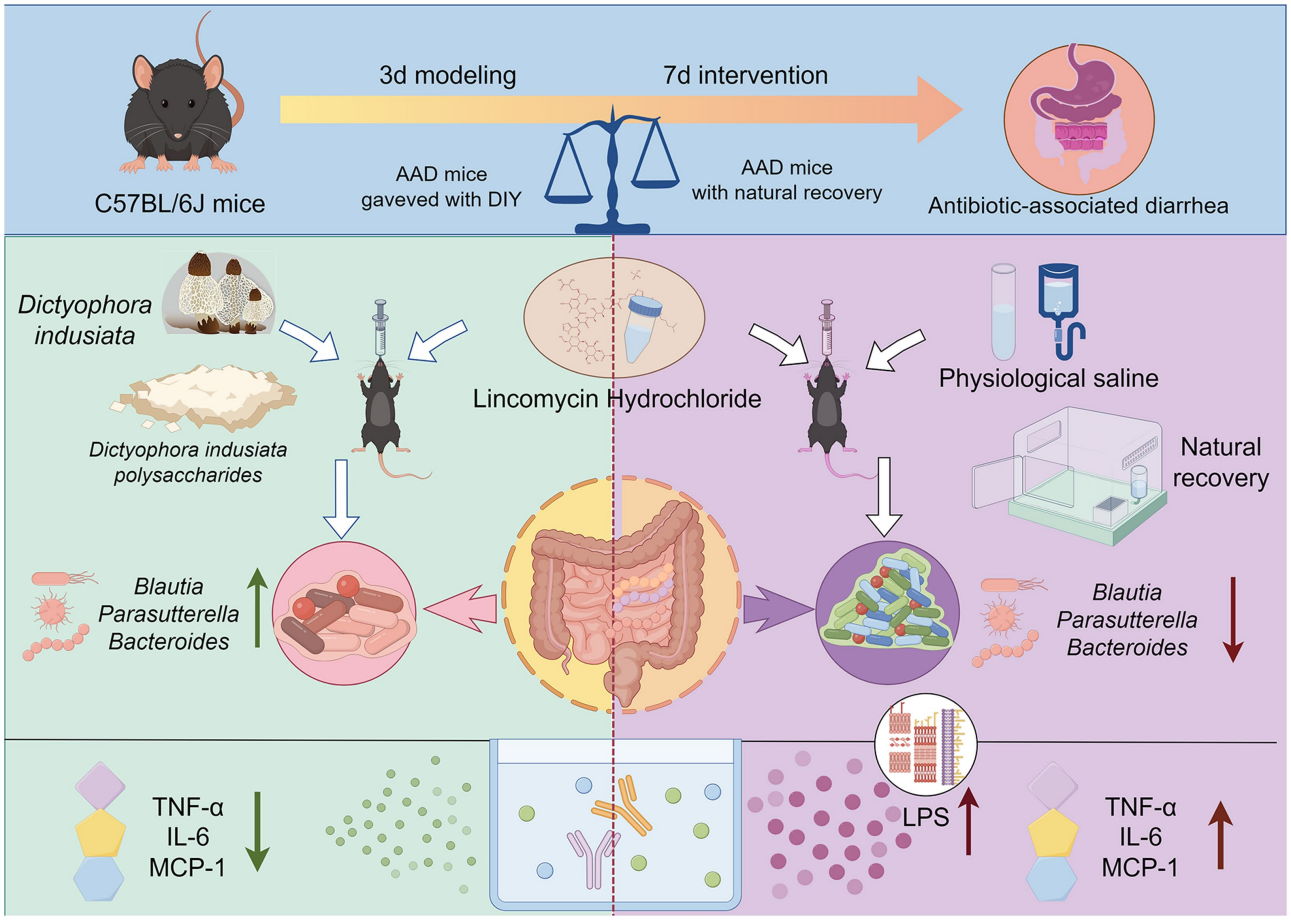
The effect and mechanism of DIPY in alleviating antibiotic associated diarrhea.

In conclusion, we have depicted the possible mechanisms by which DIPY relieves AAD that can be revealed by this experiment, as shown in the Figure 7, our results suggest that DIPY modulates intestinal flora by increasing microbial diversity and increasing the number of *Parasutterella* and *Blautia*. Meanwhile, DIPY increased the production of acetic acid, decreased the levels of LPS, MCP-1, TNF-*α*, and IL-6, and attenuated the inflammatory response, which effectively ameliorated the AAD in mice. This study confirmed that DIPY has intestinal prebiotic functions and can effectively improve AAD symptoms, providing a basis for the further development of functional products for the treatment of AAD.

## Data Availability

The original contributions presented in the study are publicly available. This data can be found at: https://www.ncbi.nlm.nih.gov, accession number PRJNA1007402.
