# Supplementary material for: Restoration of antibiotic associated diarrhea induced gut microbiota disorder by using Dictyophora indusiata water-insoluble polysaccharides in C57BL/6J mice
Source: Front Nutr. 2025 Jul 10;12:1607365. doi: 10.3389/fnut.2025.1607365 (PMC12286944; doi:10.3389/fnut.2025.1607365)
Supplement: Supplementary file 1 [file Supplementary_file_1.docx]

**Restoration of antibiotic associated diarrhea induced gut microbiota disorder by using** ***Dictyophora indusiate* water-insoluble polysaccharides in C57BL6 mice**

Yong Lai^1,3,#^, Qifan Zhang^1,#^, Maohan Xu^1^, Xiurong Guo^1^, Quan Zhou^1^, Qiuyu Liu^1^, Huiling Deng^2,^*, Can Song^1,^*

1. School of Pharmacy, Southwest Medical University, Luzhou, 646000, Sichuan, China

2. Chongqing Key Laboratory of Prevention and Treatment for Occupational Diseases and Poisoning, The First Affiliated Hospital of Chongqing Medical and pharmaceutical College, Chongqing, 400060, China

3. Institute of Traditional Chinese Medicine, Sichuan Academy of Chinese Medicine Sciences, Chengdu, 610000, Sichuan, China

* Correspondence: huiling1225@swu.edu.cn (Huiling Deng), cansong@swmu.edu.cn (Can Song)

# These authors contributed equally to this work.

**Table S1.** The α-diversity of microbial communities in mice.

| **Sample/Estimators** | **Ace** | **Chao** | **Shannon** | **Simpson** | **Coverage** | **Sobs** |
| --- | --- | --- | --- | --- | --- | --- |
| CN_1 | 219.5709 | 218.3125 | 3.351068 | 0.106293 | 0.999853 | 218 |
| CN_2 | 348.6497 | 344.9565 | 3.83987 | 0.041917 | 0.999647 | 344 |
| CN_3 | 468.0098 | 458.725 | 4.088499 | 0.053189 | 0.999177 | 454 |
| CN_4 | 308.4651 | 304.8824 | 4.050644 | 0.03349 | 0.999706 | 304 |
| CN_5 | 329.6952 | 329.0152 | 3.959729 | 0.042592 | 0.999941 | 329 |
| CN_6 | 287.9113 | 284.1 | 3.576305 | 0.062113 | 0.999647 | 283 |
| CN_7 | 245.6566 | 242.8571 | 3.62947 | 0.053666 | 0.999735 | 242 |
| NR_1 | 19 | 19 | 1.468509 | 0.313683 | 1 | 19 |
| NR_2 | 36.06875 | 35.16667 | 2.145884 | 0.155858 | 0.999941 | 35 |
| NR_3 | 28.942 | 27.6 | 2.023542 | 0.178229 | 0.999912 | 27 |
| NR_4 | 37.38509 | 37 | 2.236848 | 0.152722 | 0.999971 | 37 |
| NR_5 | 29.41667 | 29 | 1.943018 | 0.229853 | 0.999971 | 29 |
| NR_6 | 38.15863 | 36.375 | 1.674875 | 0.278131 | 0.999912 | 36 |
| NR_7 | 49.83816 | 49.125 | 2.032909 | 0.246223 | 0.999941 | 49 |
| DIPY_1 | 57.65195 | 57.11111 | 2.385436 | 0.124245 | 0.999941 | 57 |
| DIPY_2 | 76.88382 | 74.46154 | 2.745461 | 0.096316 | 0.999882 | 74 |
| DIPY_3 | 50.29217 | 50 | 2.574559 | 0.09685 | 0.999971 | 50 |
| DIPY_4 | 75.35362 | 74.25 | 2.480346 | 0.111494 | 0.999912 | 74 |
| DIPY_5 | 83.11737 | 81.58824 | 2.681286 | 0.096364 | 0.999853 | 81 |
| DIPY_6 | 82.86083 | 78.58333 | 2.599635 | 0.110107 | 0.999677 | 74 |
| DIPY_7 | 65.42465 | 64.75 | 2.699667 | 0.098482 | 0.999882 | 64 |


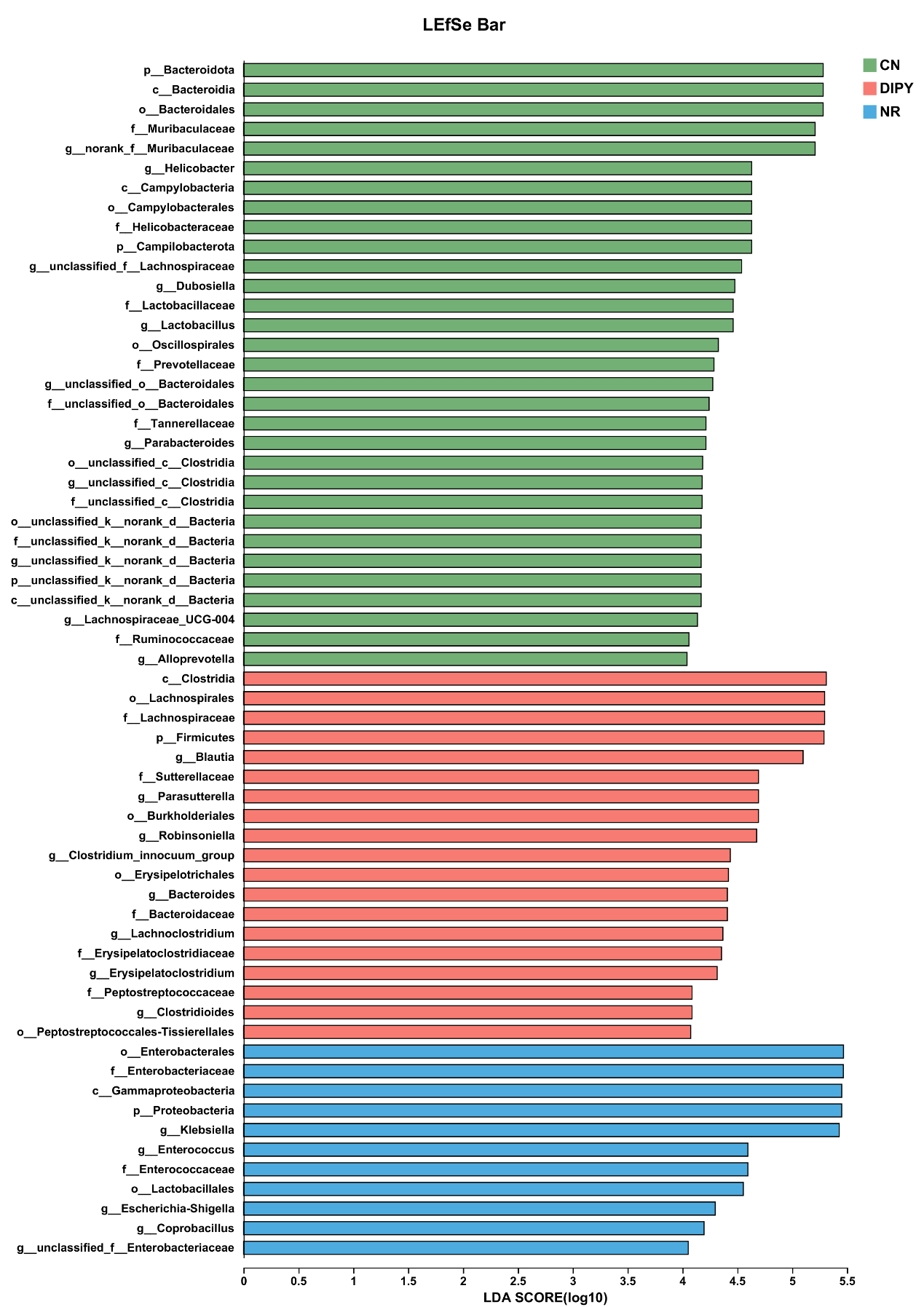


**Figure S1.** The bar chart of the results of LEfSe analysis.

**
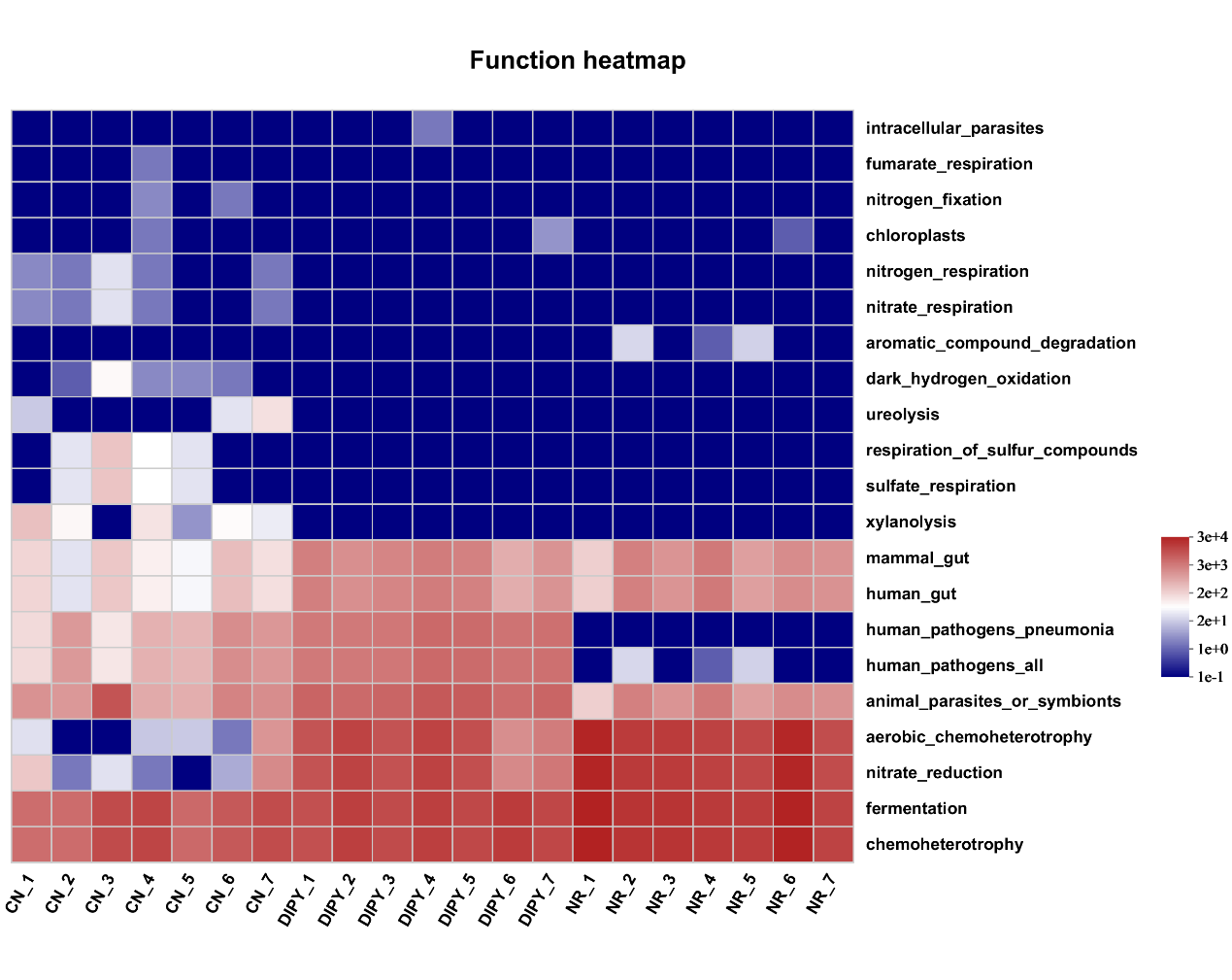
**

**Figure S2.** The heat map of the functional prediction based on FAPROTAX

**
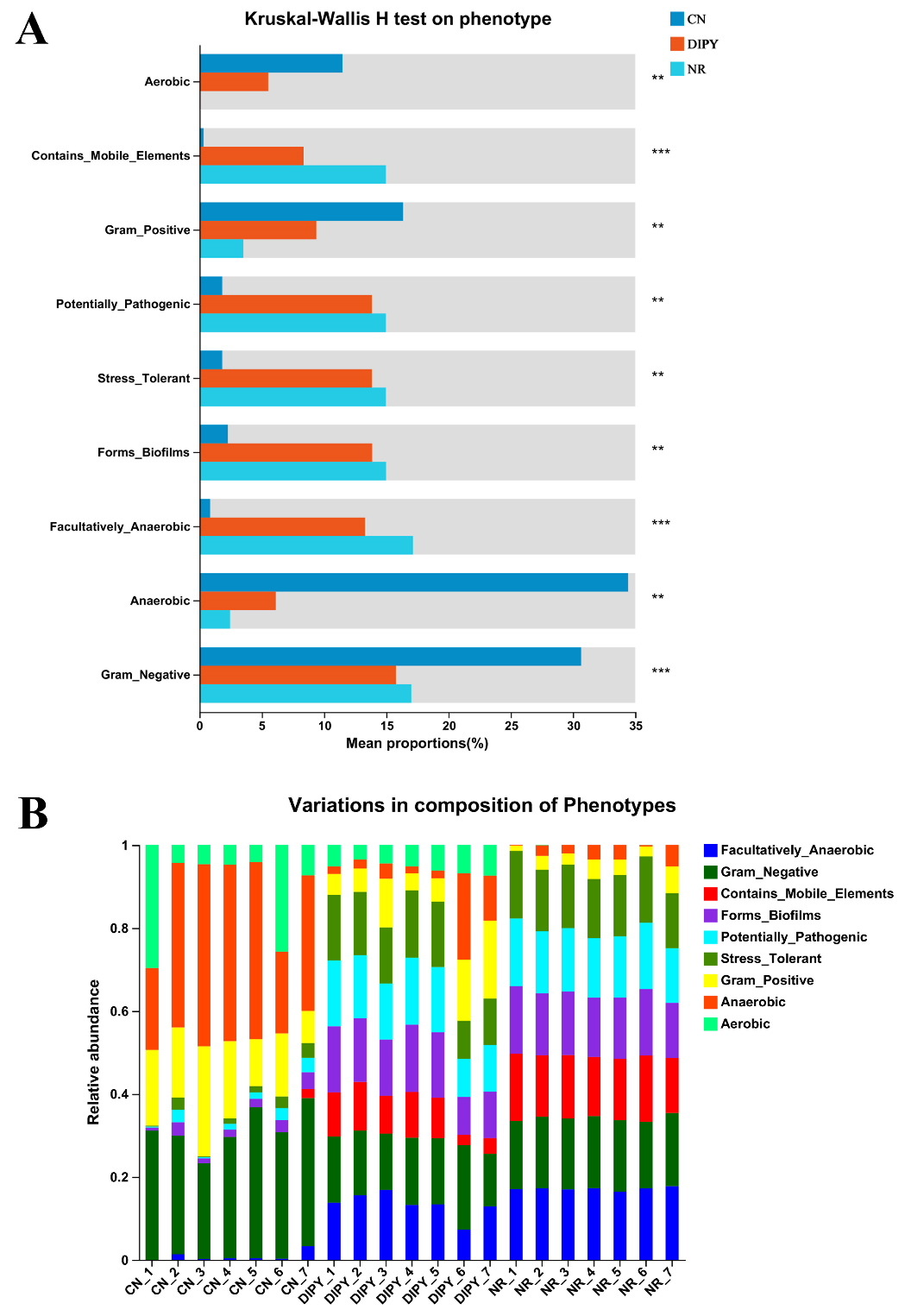
**

**Figure S3.** (A) The result of Kruskal-Wallis H test on phenotype. (B) The bar chart of the variations in the composition of phenotypes

**
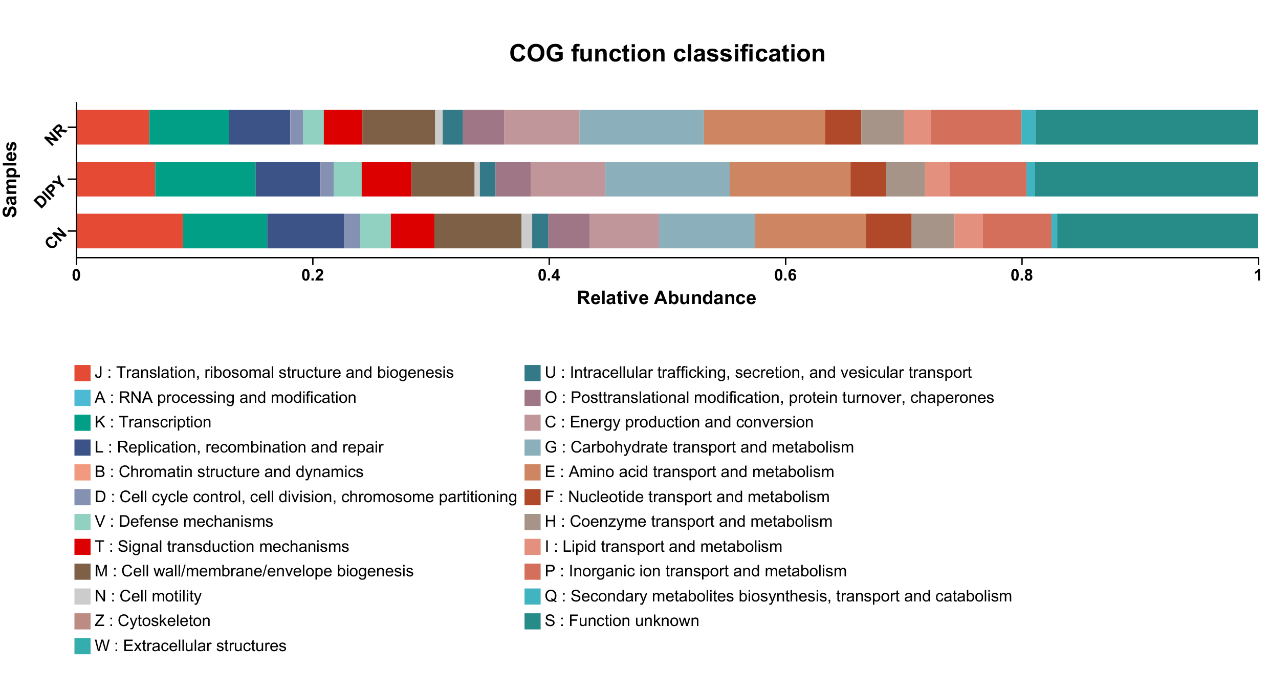
**

**Figure S4.** The COG function classification result.
